# Is There a Relationship Between Secondary Metabolites of Leaves and the Growth of *Eucalyptus* Species?

**DOI:** 10.1111/ppl.70898

**Published:** 2026-04-27

**Authors:** Ana Carina da Silva Cândido Seron, Dthenifer Cordeiro Santana, Izabela Cristina de Oliveira, Larissa Pereira Ribeiro Teodoro, Cid Naudi Silva Campos, Gileno Brito de Azevedo, Rita de Cássia Félix Alvarez, Regimar Garcia dos Santos, Paulo Eduardo Teodoro

**Affiliations:** ^1^ Federal University of Mato Grosso do Sul Mato Grosso do Sul Brazil; ^2^ University of Georgia Tifton Georgia USA

**Keywords:** daidzein, daidzin, diameter at breast height, genistein, genistin, plant height

## Abstract

Flavonoids are essential molecules that affect plant growth and development and act as defense mechanisms against biotic and abiotic factors. This study aimed to assess whether the content of flavonoids in different species of *Eucalyptus* is related to tree growth. The objective of the work is (i) to identify whether there is a relationship between flavonoids, height and diameter among *Eucalyptus* species and (ii) to identify differences in flavonoid content between species. The experimental design was randomized blocks with three replicates, with 20 plants in each experimental plot. The treatments consisted of six *Eucalyptus* species: *Eucalyptus camaldulensis* , *E. saligna* , *E. grandis* , *E. urophylla*, *C. citriodora* and a GG100 clone (hybrid clone of *E. urophylla × E. grandis*). This study measured plant diameter at breast height (DBH) and height (HC). Leaf samples were taken from the species in their respective repetitions for flavonoid evaluation, and subsequent separation and quantification of the isoflavones, daidzein (D1), daidzin (D2), genistein (G1), genistin (G2), using liquid chromatography. In general, the *E. grandis* species showed the best results for DAP and AP. D1 was higher than what? for *C. citriodora* and *E. saligna* . D2 was higher than what? for *E. camaldulensis* . The principal component analysis demonstrated a negative relationship among the variables AP and DAP with D1, D2, G1, and G2. With the results of this study, it can be inferred that there is no relationship between height, diameter, and flavonoids among the *Eucalyptus* species evaluated since the species with the lowest concentrations of isoflavones had the highest growth and diameter.

## 1 Introduction

*Eucalyptus* (family Myrtaceae) is a genus native to Australia and comprises around 700 species that are highly adapted to many environmental constraints, such as severe saline and drought conditions (Maaloul et al. 2019). *Eucalyptus* species are used for various applications, such as paper, pulp, energy generation, and charcoal, among others (da Silva et al. 2021). *Eucalyptus* stands are among the most productive forest systems globally. In Brazil, these plantations are prioritized for commercial timber production as a primary strategy to mitigate the pressure on native forests. Furthermore, this approach optimizes bioenergy conversion processes while minimizing broader environmental and socioeconomic impacts (Borges et al. 2022; da Silva et al. 2023).

The economic importance of *Eucalyptus* is associated with its distribution across a wide range of land‐use systems and agricultural ecosystems (Desta et al. 2023). Farmers are currently replacing their land with *Eucalyptus* species due to its tolerance to fire damage, resilience to losses caused by insects and grazing animals, and its efficiency as a consumer and converter of nutrients. Additionally, *Eucalyptus* exhibits high yields per hectare due to short crop rotations (Desta et al. 2023; Molla et al. 2023). If planted correctly, *Eucalyptus* species have the potential to positively impact soil physicochemical properties. Additionally, they can serve as a protective cover for crops (Khan et al. 2020).

The choice of species is a significant determinant of success in the establishment of high‐yield forests. Each species possesses distinctive characteristics and demonstrates varying degrees of adaptation to soil and climate conditions (Çiçekler et al. 2023). Significant differences in chemical composition can be observed not only between the wood of different species but also within the same species. These differences are mainly due to factors such as age, genetic diversity, and environmental influences (Terzopoulou and Kamperidou 2022). Diameter is one of the most important tree measurement variables for quantifying volume, assessing biomass and growth, and is a reference for distinguishing between thin and thick trees. In addition to diameter, another important variable is height, as it is fundamental for estimating volume and can serve as an indicator of site quality (da Rocha et al. 2023). The importance of height is paramount, as it is the basis for *Eucalyptus* productivity and species selection. Not all species are similar in their production on a given site due to factors such as soil characteristics and topography (Dean 2023).

*Eucalyptus* species are known to be a rich source of bioactive compounds and secondary metabolites, including phenolics, flavonoids, terpenoids, tannins, phloroglucinol, and cardiac glycosides, which have potential antimicrobial, antioxidant, anticancer, antiseptic, and anti‐inflammatory activities and can be used in folk medicine for a variety of medical conditions (Abdelkhalek et al. 2020; Ashour et al. 2023). Lacking motility, plants have evolved a sophisticated array of secondary metabolites to complement their innate immune systems. These specialized compounds function as critical defense compounds against herbivory and pathogen attacks, while simultaneously mitigating the constraints of abiotic stressors. Consequently, this chemical plasticity is fundamental for environmental adaptation and long‐term survival in fluctuating ecosystems (Manea et al. 2022). Flavonoids are an important group of plant secondary metabolites, widely studied in plant root exudates (Lagrange et al. 2001; Mierziak et al. 2014). Flavonoids are essential molecules that affect plant growth and development, as well as acting in plant defense mechanisms against biotic and abiotic factors (Treutter 2006). Furthermore, specific compounds from the flavone and isoflavone subgroups have been shown to protect several plant species against insect pests, influencing their behavior, growth, and development (Mierziak et al. 2014). Isoflavones, a distinct subclass of flavonoids, are typically categorized into two primary chemical forms: aglycones and their corresponding glycosidic conjugates. The aglycone group includes daidzein, genistein, and glycitein, whereas the glycosylated forms, daidzin, genistin, and glycitin, represent the predominant storage state of these metabolites in plant tissues (Abdelkhalek et al. 2020; Zhao et al. 2020).

Secondary metabolites, although not directly related to primary growth, significantly influence plant defense and their ability to adapt to adverse environmental factors. Understanding how the levels of these metabolites vary between species allows us to identify biochemical markers that can be used to select more resistant and adaptable genotypes. Obtaining knowledge of the relationship between growth and flavonoid synthesis? Can provide new strategies for genetic improvement, aiming to develop more vigorous trees with greater resilience and better performance in different environmental conditions. This study aims to evaluate the flavonoid content of different *Eucalyptus* species and relate it to tree growth to identify production patterns that contribute to more sustainable agriculture.

## 2 Materials and Methods

### 2.1 Location and Implementation of the Experiment

The experiment was carried out in the experimental area of the University of Mato Grosso do Sul, Campus Chapadão do Sul, with six species of *Eucalyptus*. The altitude of the municipality is 820 m, and the soil is classified as a medium‐textured red latosol. The region's climate, according to Köppen, is tropical humid (Aw), with two well‐defined seasons: the rainy season from October to April and the dry season between May and September. Average rainfall ranges from 750 to 1800 mm year^−1^ and the average annual temperature ranges from 20°C to 25°C.

Soil management at the site was carried out by the needs of the area according to the chemical analysis of the soil, with the following results: pH (CaCl_2_): 4.9; organic matter: 31.5 g dm^3^; phosphorus: 13.6 mg dm^3^; hydrogen + alum (H + Al): 5.4; potassium: 0.29 cmol dm^3^; calcium: 2.8 cmolc dm^3^; magnesium: 0.5 cmolc dm^3^; cation exchange capacity (CEC): 9.0 cmolc dm^3^; base saturation: 39.9%. The proportions of clay, sand, and silt were 46%, 46%, and 8%, respectively. Weed management was performed by mechanical mowing every 60 days, followed by the application of the glyphosate herbicide at a rate of 4 l ha^−1^ of commercial product.

The experimental design was randomized blocks with three replicates. Each plot consisted of 20 plants. The treatments consisted of six different species of *Eucalyptus*: *E. camaldulensis* , *E. urophylla*, *E. saligna* , *E. grandis* , *C. citriodora* , and a GG100 clone (*E. urophylla* × *E. grandis* hybrid clone).

### 2.2 Variables Evaluated

The data used in this study were obtained throughout 2023 by measuring the diameter at breast height (DBH) and total height (Tt) in two seasons: March and May, with 82 and 88 trees, respectively; the trees were about 10 years old. To obtain the DBH (cm), a tape was used to measure the circumference at breast height, which was converted to DBH, and the total height (m) was obtained using a Haglof hypsometer.

Leaf samples were taken from the species in their respective repetitions for flavonoid evaluation by liquid chromatography. To extract the isoflavones, 50 mg of the samples were added to a 2 mL Eppendorf tube, with 1.5 mL of 70% methanol containing acetic acid (0.1%). The mixture was stirred briefly and then incubated for 2 h in ultrasound. Subsequently, the samples were centrifuged at 935*g* for 20 min at 4°C (Kasvi K14‐4415R). The supernatant obtained was filtered using a syringe with a 0.2 μm filter and transferred to 1.5 ml vials before injection into an ultra performance liquid chromatography (UPLC) system, model ACQUITY UPLC H‐Class (Waters). Aliquots of 10 μL were used for direct injection into the UPLC. All the analytical procedures were performed following the methodology described by de Oliveira et al. (2024).

Isoflavone identification was performed using commercial standards of daidzein, genistein, genistin, and daidzin. The qualitative and quantitative profiles of the chromatographic peaks were confirmed by comparing retention times and UV spectra with those of individual standards, further validated by the standard addition method (Carrão‐Panizzi et al. 2002). Detection was conducted using a Waters photodiode array (PDA) detector at a fixed wavelength of 254 nm.

All the solvents used in the chromatographic analysis were HPLC grade (Merck), and before use, they were vacuum filtered through a 0.2 μm pore membrane and then degassed in a vacuum system using ultrasound. The water used was distilled and then ultra‐purified in a Milli‐Q system and then degassed.

### 2.3 Statistical Analysis

The variables Ht, DAP, D1, D2, G1, and G2 were analyzed using species as a fixed effect and seasons as a random effect. If species were significant, the means were grouped using the Scott–Knott test at 5% probability. The sisvar software was used for these analyses.

The data were also subjected to principal component analysis (PCA) to assess the relationship between the variables and the species. Pearson's correlation analysis was also carried out to check the interdependence between the variables analyzed within each species. Both graphs were made using RStudio software.

## 3 Results

The analysis highlighted significant variations between the species in the variables Ht, DAP, D1, and G1. In addition, there were differences related to the Season factor, which were notable in D1, D2, and G1. The interaction between species and season revealed significant effects on D1 and G1. However, because the season factor was considered random, it was decided to disregard both the season factor and the species × season interaction (Table 1).

**TABLE 1:** Summary of the analysis of variance for the variables plant height (Ht), diameter (DBH) and for the isoflavones: Daidzein (D1), daidzin (D2), genistein (G1), and genistin (G2) evaluated in six *Eucalyptus* species in two evaluation periods.

F.V.	G.L.	Ht	DBH	D1	D2	G1	G2
Block	2	16.55^ns^	0.52^ns^	17,574^ns^	355,191^ns^	1277^ns^	9,395,290 ^ns^
Species	5	1305.38*	131.66*	103,158*	369,175^ns^	1,428,885*	558,743^ns^
Season	1	17.20^ns^	14.71^ns^	809,163*	37,129,056*	2,275,351*	539,635^ns^
Species × season	5	8.14^ns^	1.782^ns^	88,917*	270,991^ns^	127,284*	5,955,439^ns^
Residuals	22	33.71	4.43,1364	9110	596,343	18,738	2,939,024

*Note:* F.V.—source of variation; G.L.—degrees of freedom.

* Significant at 5% probability by the *F* test.

The analysis of variance revealed different patterns for the variables studied in the six *Eucalyptus* species (Table 2). For the plant height variable (Ht), it was noted that the *E. grandis* species had the highest values, followed by GG100, *E. urophylla*, and *E. saligna* , while *C. citriodora* and *E. camaldulensis* had the lowest values, respectively. In terms of diameter (DBH), *E. grandis* and GG100 stood out with the highest values, followed by *E. urophylla* and *E. saligna* , while *C. citriodora* and *E. camaldulensis* had the lowest values. The concentration of daidzein (D1) was highest in *C. citriodora* and *E. saligna* , followed by *E. urophylla* and *E. camaldulensis* . GG100 and *E. grandis* recorded the lowest concentrations of this isoflavone. In the case of genistein (G1), the species *E. camaldulensis* had the highest values, followed by *E. grandis* , while *E. urophylla*, GG100, *E. saligna* , and *C. citriodora* showed the lowest values.

**TABLE 2:** Grouping of means for the following variables: Plant height (PH), diameter (DBH), daidzeín (D1), and genisteín (G1) assessed in six *Eucalyptus* species in two assessment periods.

Species	Ht (m)	DBH (cm)	D1 (mg 100 g MS)	G1 (mg 100 g DM)
*E. camaldulensis*	43.26 d	16.29 d	250.63 b	544.45 a
*C. citriodora*	52.53 c	18.90 c	325.08 a	229.52 c
*E. saligna*	69.89 b	25.15 b	371.83 a	206.13 c
*E. urophylla*	70.99 b	23.59 b	220.55 b	109.43 c
GG100	72.53 b	27.70 a	57.16 c	196.66 c
*E. grandis*	83.55 a	27.63 a	63.22 c	353.23 b

*Note:* Means followed by the same letters in the same column belong to the same group by the Scott–Knott test at 5% probability.

**FIGURE 1:**
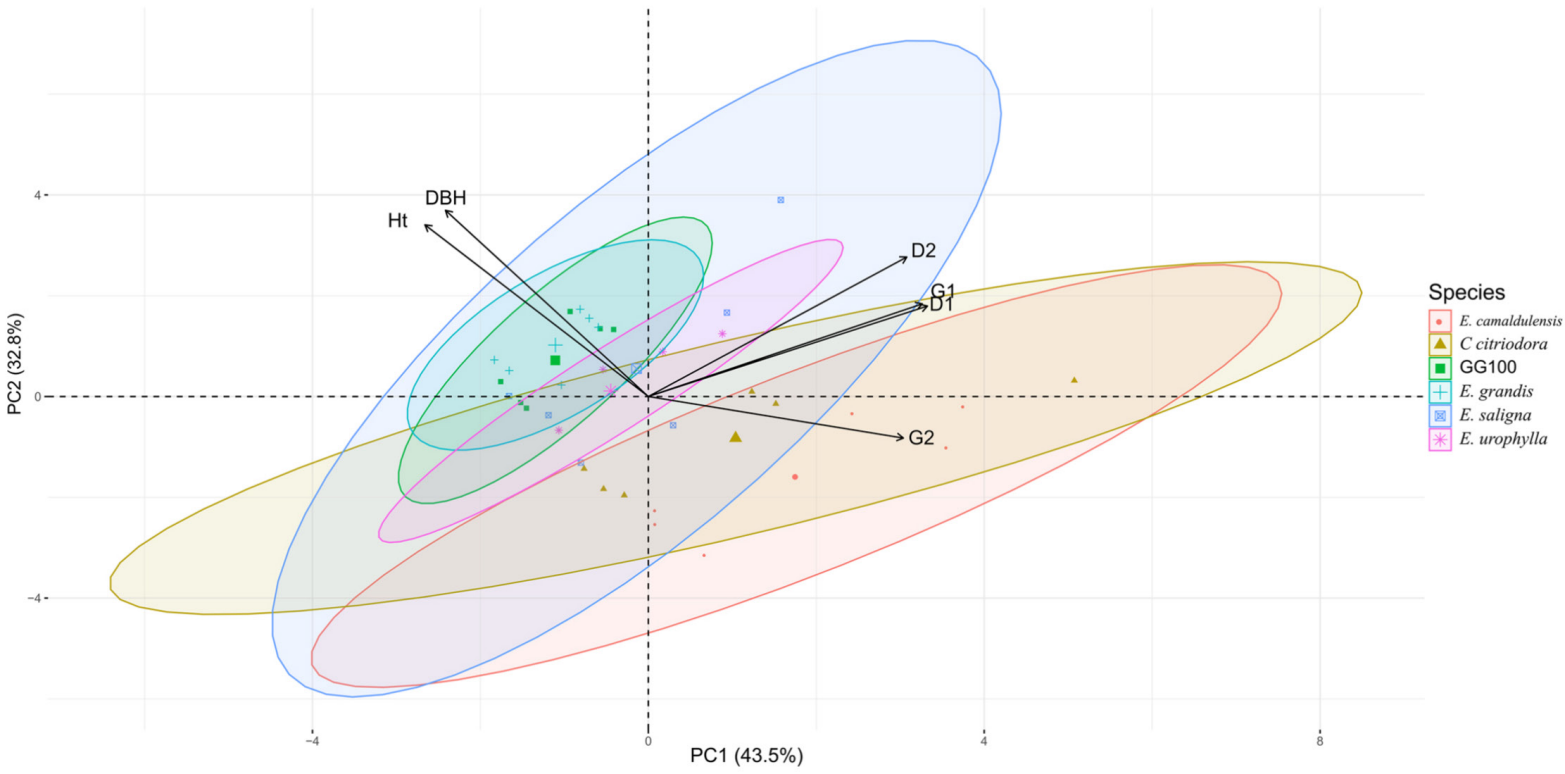
Principal components (PCA) to observe the relationship of *Eucalyptus* species with the variables plant height (Ht), diameter (DAP), and for the isoflavones: Daidzein (D1), daidzin (D2), genistein (G1), and genistin (G2).

PCA showed that the first component explains 43.5% of the variability in the data, while the second component explains 32.8% (Figure 1). Considering that the resulting two‐dimensional graph covers 76.3% of the total variability of the data, it can be said that this is appropriate for assessing the relationships between the variables. The two‐dimensional plot of the principal components revealed a negative correlation between the parameters Ht and DAP with the parameters D1, D2, G1, and G2. Notably, the species GG100 and *E. grandis* exhibited higher values for Ht and DAP, while they showed lower values for D1, D2, and G1. On the other hand, the species *C. citriodora* and *E. camaldulensis* showed lower values for Ht and DAP, but higher values for D1, D2, G1, and G2.

**FIGURE 2:**
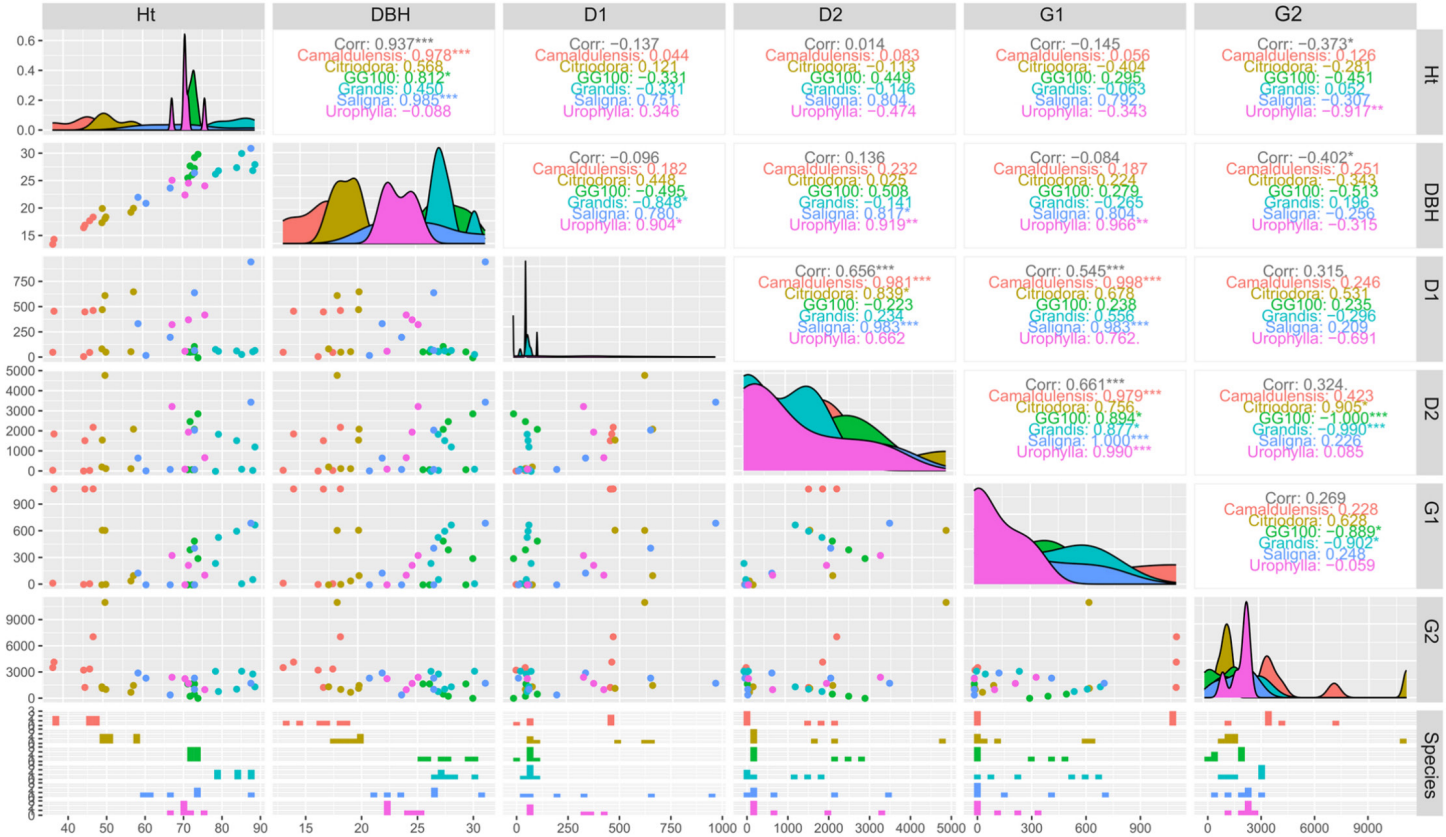
Pearson's correlation matrix illustrating the relationships between plant height (AP), diameter at breast height (DAP), and isoflavone concentrations (daidzein [D1], daidzin [D2], genistein [G1], genistin [G2]) across different *Eucalyptus* species. Diagonal panels: Display kernel density estimates for each variable, showing their distribution. Upper triangle panels: Present Pearson's correlation coefficients (*r*) between variable pairs, disaggregated by *Eucalyptus* species. Asterisks indicate statistical significance (*p* < 0.05, *p < 0.01, **p < 0.001). Lower triangle panels: Illustrate scatter plots for each pair of variables, depicting their bivariate relationships. Rightmost column panels: Show box plots for each variable, comparing their distributions across the evaluated *Eucalyptus* species.

Pearson's correlation analysis was carried out to investigate the interrelationships between the variables, including plant height (Ht), diameter (DAP), and isoflavones (D1, D2, G1, G2), in each *Eucalyptus* species examined, as shown in Figure 2. Regarding plant height (Ht), there was a positive relationship between plant height (Ht) and diameter (DBH) in the *E. camaldulensis* , GG100, and *E. saligna* species, indicating that an increase in one variable is associated with an increase in the other. On the other hand, a negative correlation was identified between Ht and G2 in the *E. urophylla*, suggesting that an increase in Ht is related to lower values in G2. No significant correlations were identified between Ht and the variables D1, D2, and G1.

For diameter (DBH), there was a positive correlation with D1, D2, and G1 in the *E. urophylla* species, while G2 showed a negative correlation, indicating that higher DAP values are associated with lower G2 values in the species analyzed. In the case of the D1 variable, there was a positive correlation with D2 in the *E. camaldulensis* , *C. citriodora* , and *E. saligna* species, as well as a positive correlation with G1 in *E. camaldulensis* and *E. saligna* . When exploring the G1 variable, a positive correlation was identified with G2 in the GG100 and *E. grandis* species, highlighting an association between these two variables in these specific species.

## 4 Discussion

DBH is a variable that is easy to obtain and has a high correlation with volume and height, making it an independent and essential piece of data (Kainer et al. 2018; de Oliveira et al. 2021). However, total height and diameter at breast height are the two main parameters used to calculate the basal area and volume in a forest (Viera et al. 2013; Figueiredo et al. 2020).

The slight variation between heights is due to the homogeneity of the environmental characteristics of the region, such as the same soil and consequently the same location index (Calegario et al. 2005; Soares et al. 2016). The direct and positive relationship between diameter and height is widely reported in studies (Schönau and Coetzee 1989; Goktas et al. 2023; Hua et al. 2023). Various studies carried out to assess the growth of *Eucalyptus* species in different ecological regions show that, for the same species, performance varies depending on the growing site (Del Quiqui et al. 2001; Coutinho et al. 2004). The study of DBH and Ht has several applications, such as making it possible to infer the productive capacity of the plantation, in addition to contributing to the selection of trees with better characteristics suitable for each location (Kainer et al. 2018; de Oliveira et al. 2021). Therefore, it is important to investigate the relationship between the concentration of these compounds and tree growth, identifying physiological adaptations that impact development, especially under adverse conditions. Observing these relationships in already developed plants can provide information that aims to improve plant establishment and growth while still in the seedling stage, contributing to genetic improvement strategies that seek to increase plant resilience to stress factors.

Secondary metabolites, such as flavonoids, are not directly involved in primary growth processes, but they play essential roles that can indirectly affect tree vigor and health. The content of isoflavones varies greatly from one species to another. Some studies have reported that as species grow, they may have lower isoflavone levels and that biotic and abiotic stress factors affect the roots, increasing isoflavone concentrations (Mustonen et al. 2018).

Polyphenolic compounds, phenolics, and flavonoids play essential roles in the resistance of plants against pathogenic microorganisms (Abdelkhalek et al. 2020). Flavonoids are compounds found in plants that have the following properties: antioxidant, bactericidal, antifungal, antiviral, and anti‐inflammatory; in addition, they are molecules that protect plant material in its environment against microorganisms, UV rays, and herbivores, and are also the pigment responsible for the coloring of various plant species (Vizzoto et al. 2010). A direct correlation exists between leaf flavonoid concentration and the market value of the raw material. This relationship underscores the strategic importance of phytochemical characterization for the pharmaceutical industry, which prioritizes high‐quality biomass to optimize the production of bioactive compounds (Zheng and Wang 2001; Trevizan et al. 2023).

Isoflavonoids have enormous potential for fighting a range of diseases. Isoflavones, such as genistein and daidzein, are commonly considered phytoestrogens due to their estrogenic activity (Diwan et al. 2017). Genistein is classified as the isoflavone with the highest estrogenic activity in vivo, being around 10 times more active than daidzein (Branham et al. 2002).

According to the PCA, no relationship was observed between DAP, Ht and flavonoid levels in the species studied. In general, the species that showed a greater relationship with growth variables did not show a relationship with secondary metabolites, and the reverse was also true. Of note was the species *E. urophylla*, which demonstrated a certain association between secondary metabolites and growth variables. These results suggest that, for most species, growth, measured by DAP and Ht, is influenced by factors other than secondary metabolites. This relationship may be due to the fact that flavonoids, as defense and signaling compounds, play specific roles in adaptation to stresses and do not necessarily correlate directly with growth indicators under normal conditions (Chen et al. 2022). In the case of *E. urophylla*, the observed relationship may indicate an adaptive response; flavonoids contribute to growth by mediating tolerance to environmental stresses, which would justify this relationship.

Furthermore, correlations suggest that although there are some associations between growth and isoflavones in certain species, these compounds may play distinct roles in regulating tree growth and adaptation. Secondary metabolites may not be the main determinants of growth under the conditions analyzed, but their study may reveal important information about the adaptation of species to environmental stresses. These findings highlight the need for additional studies to understand the mechanisms by which flavonoids and other secondary metabolites influence growth in different contexts and species.

Based on the results obtained in this study, it is suggested that future research should be carried out to determine the concentration of each type of flavonoid present in *Eucalyptus* leaves and correlate it with the variables height and DBH since it is not yet clear what role the type of flavonoid plays in this relationship. In addition, it is essential to develop genetic improvement programs to obtain trees with greater productivity and efficiency in the accumulation of these isoflavones since the identification of plants that are more efficient in the composition of these flavonoids suggests that these plants have potential production for drugs that treat a range of diseases.

## 5 Conclusions

Analysis of variance showed distinct patterns among the six *Eucalyptus* species. *E. grandis* and GG100 presented the highest height and diameter values, while *C. citriodora* and *E. camaldulensis* had the lowest. Regarding the compounds, *C. citriodora* and *E. saligna* stood out with the highest concentrations of daidzein, and *E. camaldulensis* had the highest levels of genistein. These results reflect genetic and adaptive variations important for forest improvement.

The results showed that the relationships between growth, height and diameter and flavonoid compounds vary according to the *Eucalyptus* species. Principal component and correlation analyses confirmed the absence of a consistent association between flavonoids and growth in most species, except in *E. urophylla*, which presented a differentiated pattern. These findings indicate that secondary metabolites, although not the main determinants of growth, can influence specific adaptive responses.

## Author Contributions

A.C.S.C.S., D.C.S. and P.E.T. wrote the main manuscript text; D.C.S., I.C.O., L.P.R.T., C.N.S.C., G.B.A., R.C.F.A. and R.G.S. conducted the experiments and performed the evaluations. All authors reviewed the manuscript.

## Data Availability

The data is available from the corresponding author upon reasonable request.
